# Mapping the Interactions Among Class IIa Histone Deacetylases and Myocyte Enhancer Factor 2s

**DOI:** 10.1021/acs.jcim.5c00858

**Published:** 2025-06-06

**Authors:** Narayan Gautam, Sophia Wang, Aykut Üren, Prem P. Chapagain, Narayan P. Adhikari, Purushottam B. Tiwari

**Affiliations:** a Central Department of Physics, 97983 Tribhuvan University, Kirtipur, Kathmandu 44613, Nepal; b Tri-Chandra Multiple Campus, 97983 Tribhuvan University, Ghantaghar, Kathmandu 44613, Nepal; c Department of Biology, Georgetown University, Washington, D.C. 20057, United States; d Department of Oncology, Georgetown University, Washington, D.C. 20057, United States; e Department of Physics, 5450 Florida International University, Miami, Florida 33199, United States; f Biomolecular Sciences Institute, 5450 Florida International University, Miami, Florida 33199, United States

## Abstract

Myocyte enhancer factor 2 (MEF2) transcription factors regulate several developmental programs, including the control of neural crest development and neuronal differentiation as well as survival. MEF2s are highly expressed in cerebellar granule neurons. Class IIa histone deacetylases (HDACs), abundantly expressed in the brain as well, repress gene expression activity of MEF2 via physical interactions and play a critical role in neuronal apoptosis. In this work, we conducted molecular dynamics (MD) simulation-based investigations to investigate interactions among different class IIa HDACs (HDAC4, HDAC5, HDAC7, and HDAC9) and MEF2s (MEF2A, MEF2B, MEF2C, and MEF2D). Our results show that hydrophobic interactions are the main mechanism for the formation of class IIa HDAC-MEF2 complexes. Our analysis shows that L66 and L67 in all MEF2s mostly contribute to the hydrophobic interactions. All residues that establish hydrophobic interactions, hydrogen bonding, and salt bridges are conserved in all MEF2s. Calculations of the MM/GBSA binding free energies also show that the class IIa HDAC-MEF2 complexes exhibit comparable binding affinities. We performed surface plasmon resonance (SPR)-based direct binding experiments using four different purified class IIa HDACs and MEF2A to validate our computational investigations. The SPR results confirmed the direct binding between the class IIa HDACs and MEF2A with fairly comparable nanomolar affinity (3.5 nM to 19.1 nM). This is a comprehensive study to map interactions among class IIa HDACs and MEF2s. We believe that our investigation offers the scientific community valuable insights to further understand, explore, and investigate biomolecular systems that include the class IIa HDAC-MEF2 complex formations.

## Graphical abstract

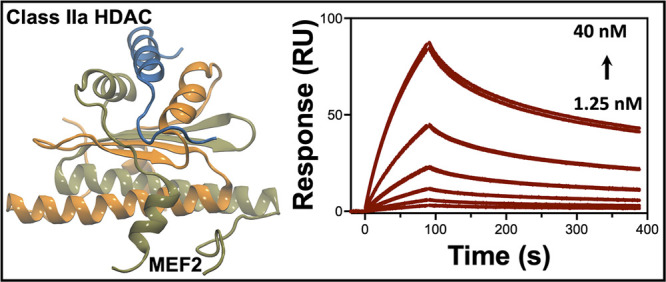

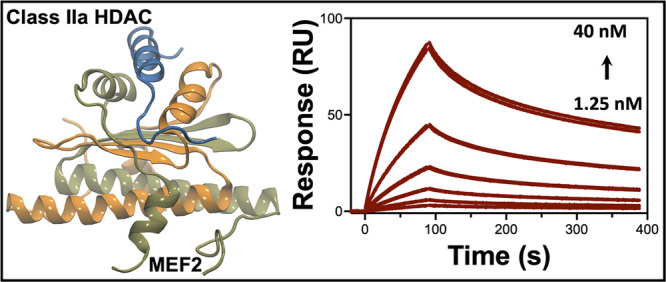

## 1 Introduction

Myocyte enhancer factor 2 (MEF2) transcription factors are highly expressed in cerebellar granule neurons (CGNs),^1^ regulate several developmental programs that include control of neural crest development, neuronal differentiation, and neuronal survival.^2–6^ MEF2A, MEF2B, MEF2C, and MEF2D are four different types of human MEF2s.^6^ MEF2s form dimer^7^ mediated by the MADS (MCM1, agamous, deficiens, SRF) and MEF2 domains.^8,9^ Histone deacetylases (HDACs) play roles in regulating different cellular processes such as cell proliferation, differentiation, and apoptosis.^10–13^ HDAC4, HDAC5, HDAC7, and HDAC9 belong to class IIa subclass of HDACs.^13,14^ These class IIa HDACs are abundantly expressed in the brain, heart, and musculoskeletal tissues.^15^ Class IIa HDACs often follow nuclear/cytoplasmic transport.^10,16,17^ Unphosphorylated class IIa HDACs are found in the nucleus and interact with transcription factors under basal conditions.^14^ Under certain cellular conditions, such as low-potassium as well as excitotoxic glutamate conditions, and even when cells are treated with certain inhibitors, there is a possibility of nuclear transport of class IIa HDACs.^18,19^ Class IIa HDACs repress MEF2-associated gene expression^20^ via physical interactions with MEF2s. In addition to myogenesis, neuronal survival, axon branching, and regulation of neuronal cell death are associated with the formation of class IIa HDAC-MEF2 complexes.^18,21,22^ Therefore, investigations leading to the formation and characterization of the class IIa HDAC-MEF2 complexes are of significant interest and implications.

In neurodegenerative diseases, neuronal protection from cell death via apoptosis is possible when the transcription activity of MEF2 is enhanced.^23^ When HDAC-MEF2 complex dissociates, the expression of prosurvival genes occurs because MEF2 activity gets retained.^23^ Class IIa HDACs regulate neuronal apoptosis,^12,21,24,25^ and their function can be inhibited by blocking interactions of the class IIa HDACs with MEF2.^26^ As such, inhibition of the class IIa HDAC-MEF2 complex formation helps develop new therapeutics to combat neurodegenerative diseases.

While the C-terminal region in all MEF2s are diverse, the vertebrate MEF2s exhibit highly conserved N-terminal regions,^7^ which are responsible for interacting with DNA and other proteins.^27^ The N-terminal domain in class IIa HDACs interacts with MEF2 transcription factors.^14^ Previous investigations suggest that a short amphipathic helix in class IIa HDACs binds to a highly conserved MEF2 hydrophobic groove located on the MADS-Box/MEF2 domain.^7,27,28^ Recently, we characterized the formation of the HDAC7-MEF2A complex.^29^ Despite invested interest and limited investigations, a complete picture of interaction among all class IIa HDACs and MEF2s has not yet been elucidated. This drew our significant interest to continue investigations to encompass all members of class IIa HDACs and MEF2s. In our recent publication,^29^ we reported that only a truncated HDAC7 portion with amino acids from position K76 to N108 was sufficient to form the HDAC7 complex with MEF2A. Our prediction revealed that the HDAC7 residues that interact with MEF2A are mostly conserved in the class IIa HDACs, and MEF2A amino acid residues that interact with HDAC7 are fully conserved in all MEF2s.^29^ Our results also predicted that recruitment of DNA to MEF2A did not show significant alteration in HDAC7-MEF2A binding.^29^ Therefore, based on our recent results,^29^ we selected only truncated class IIa HDAC regions and did not include DNA in all class IIa HDAC-MEF2 complexes in this study. We also included the crystal structures available for complexes of human class IIa HDACs with human MEF2A and MEF2D in our all-atom simulation studies. The simulation of these crystal structures serves as a control in our studies. Excluding HDAC7-MEF2A that we investigated in our recent report using the same computational approach,^29^ we predicted and studied 15 additional new class IIa HDAC-MEF2 complexes and 5 available structures, totaling 20 different complex systems. We conducted three independent 500 ns all-atom simulations of all 20 complexes. The interacting amino acid residues that we predicted are listed as the common interacting residues from the results of the three replica simulations for each complex to characterize binding interfaces among all class IIa HDACs and MEF2s.

Our results show that the hydrophobic interaction plays a major role in the formation and stabilization of the class IIa HDAC-MEF2 complexes. We found that L66 and L67 in MEF2s consistently establish hydrophobic interactions. Together with residues L66 and L67, all other residues that establish hydrogen bonding and salt bridges with class IIa HDACs are conserved in all MEF2s. Calculations of MM/GBSA binding free energies for all 15 different class IIa HDAC-MEF2 complexes that we predicted show that the interactions exhibit comparable binding affinities. Based on these results, we experimentally validated binding of all class IIa HDACs to MEF2A and determined binding affinities using the surface plasmon resonance (SPR) technique. The experimental results show that the class IIa HDACs bind to MEF2A with fairly comparable nanomolar affinity (3.5 nM to 19.1 nM). Our work, therefore, presents a comprehensive study to provide valuable insights and map interactions among functional class IIa HDAC-MEF2 complexes. We hope that this comprehensive study will be valuable to the scientific community for further scientific endeavors leading to investigations involving these functional complexes.

## 2 Results and Discussion

### 2.1 Predicted Class IIa HDAC-MEF2 Complexes and Their Stability

We performed 500 ns all-atom simulations of 15 different predicted class IIa HDAC-MEF2 complexes. We also chose complexes with human HDAC4-MEF2A, HDAC4-MEF2D, HDAC5-MEF2D, HDAC7-MEF2D, and HDAC9-MEF2D that have available crystal structures as controls, which adds to 15 predicted complexes to make a total of 20 different simulation systems. Notably, we conducted three independent replica runs for each system (60 simulation trajectories in total) and interpreted the results based on multitrajectory analyses.

Figure 1 shows the representative complex structures at the end of the 500 ns simulations. Structure files of these complexes are provided in PDB format (Supporting Information, Section S1). The class IIa HDAC-MEF2 complex structures with crystal structures at the end of 500 ns simulations are shown in Figure S1 (Supporting Information). We also performed PCA-based cluster analysis and produced average structures from the simulation trajectories of all 15 different complexes that we studied. The representative average structures are provided in the Supporting Information (Section S1). In our previous report,^29^ we predicted that only the truncated portion of HDAC7 (amino acids from position K76 to N108) was sufficient to form the HDAC7-MEF2A complex. We used the same range of the HDAC7 amino acids to prepare the simulation systems of complexes HDAC7 with MEF2B, MEF2C, and MEF2D, as we used to prepare the HDAC7-MEF2A complex that was used in all-atom simulations in our previous report.^29^

**Figure 1:**
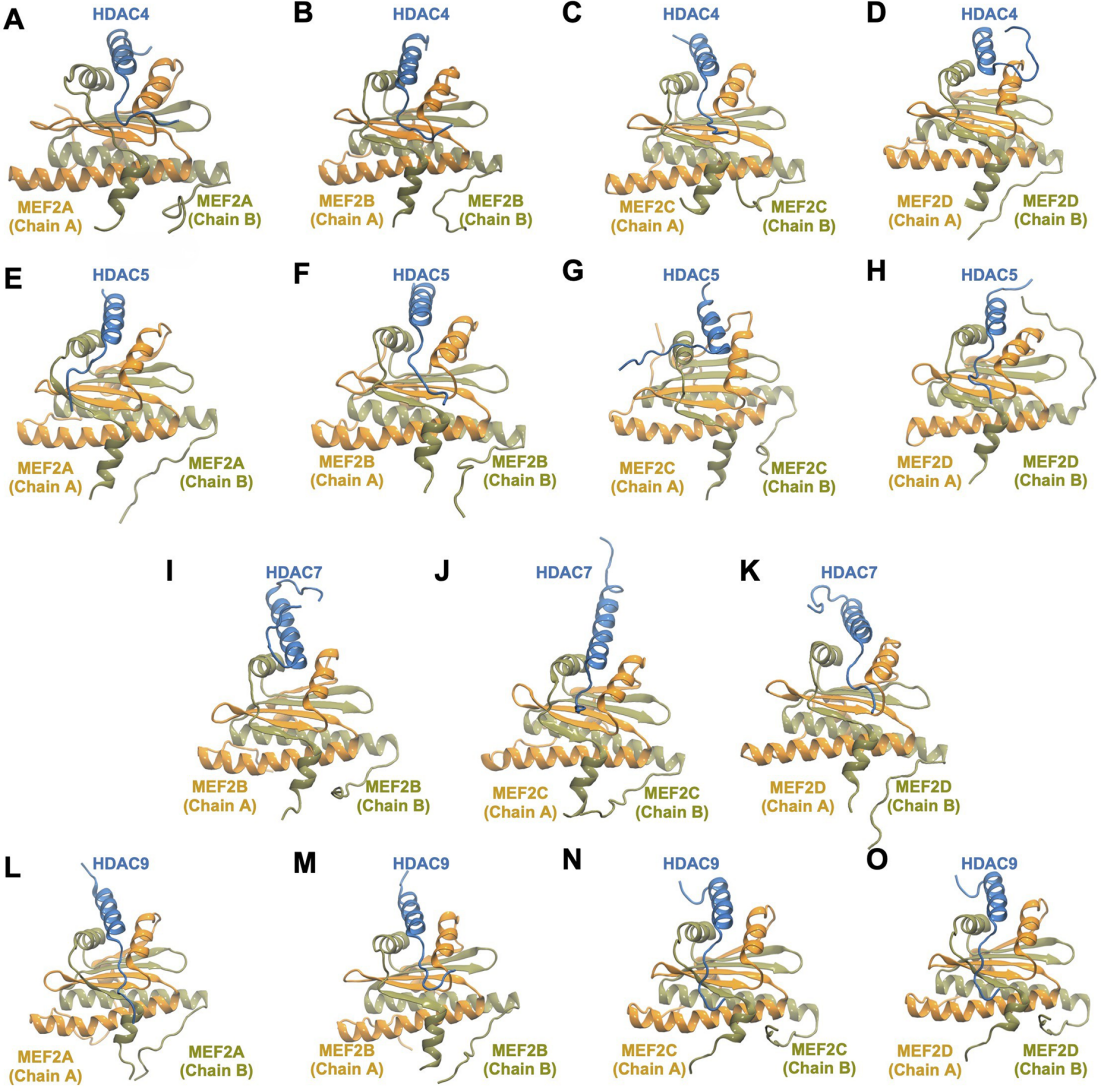
(A–O) Representative class IIa HDAC-MEF2 complex structures at the end of the 500 ns MD simulations. Structures in light blue color represent class IIa HDACs. Structures in light orange and light brown colors represent two monomers of each MEF2 dimer.

A multiple sequence alignment results in our previous report^29^ predicted that five out of six HDAC7 interacting residues were conserved in all class IIa HDACs, and the remaining one HDAC7 interacting residue is conserved in three class IIa HDACs. These alignment results provided us a guidance to select the range of amino acids that we could potentially use in the truncated HDAC4, HDAC5, and HDAC9 structures in complex with MEF2s.^29^ Moreover, the range of the HDAC7 portion that we predicted to interact with MEF2A in our recent publication^29^ is well within the range (from amino acid position 72 to 172) as outlined in a prior experimental prediction.^30^ The published crystal structures, so far until this investigation, as mentioned in Section 3.2, also presented only the truncated class IIa regions interacting with MEF2s. We thus believed that the truncated class IIa HDACs regions were sufficient for their interactions with MEF2s. We slightly extended the range of the amino acids in these three class IIa HDACs from the conserved range of amino acids corresponding to HDAC7 residues interacting with MEF2A. We used amino acids with positions from G161 to K184 for HDAC4, from S173 to K196 for HDAC5, and from G133 to K158 for HDAC9 to create simulation inputs. It is to be noted that we first generated the full-length class IIa HDAC-MEF2 complexes and confirmed that the same HDAC4/5/9 ranges as mentioned above are in close proximity to MEF2s. This truncation offered much less computational cost for each of the 45 all-atom simulations of the predicted complexes for 500 ns.

We monitored the stability of all class IIa HDAC-MEF2 complexes via measurements of the root-mean-square deviation (RMSD) and radius of gyration (*R*_g_). Figure S2A–D (Supporting Information) shows RMSD measurements, and Figure 2A–2D represents *R*_g_ measurements. Different colors represent different class IIa HDAC-MEF2 complexes. Since we repeated simulations for each class IIa HDAC-MEF2 complex three times, we have presented measurements from all three simulations in light colors with the corresponding dark color as the average values for each triplicate. The stability of RMSD and *R*_g_ curves shows that the complexes were stable during the simulations. Single runs of a couple of complexes seemed to be fluctuating but stabilized toward the end of the simulations. Figure S2 in the Supporting Information shows RMSD and *R*_g_ measurements for the complexes with available crystal structures during this investigation.

**Figure 2:**
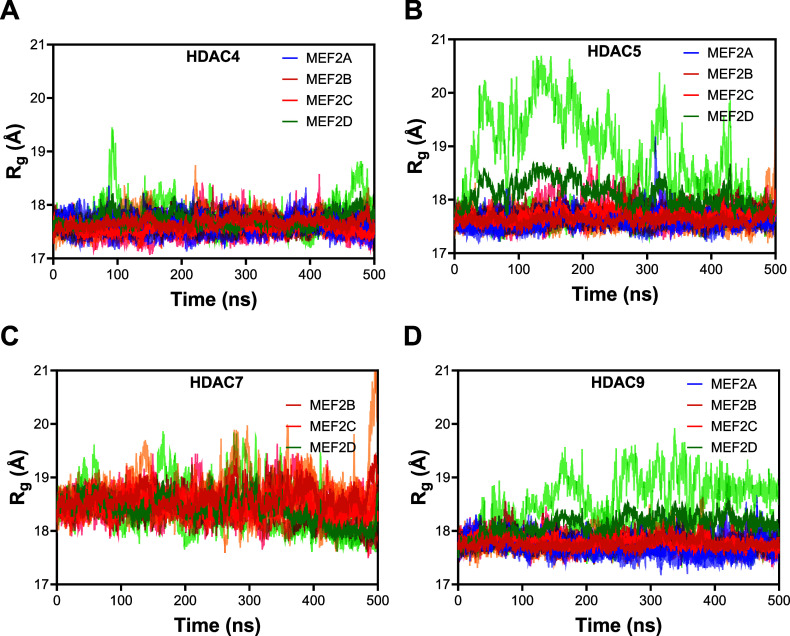
(A–D) Radius of gyration (*R*_g_) measurements for class IIa HDAC-MEF2 complexes. The same light-colored data correspond to the measurement from three different runs with the respective dark color as the average values for each triplicate.

While RMSD assesses better convergence and stable conformation and *R*_g_ corresponds to compactness of simulated structures throughout the simulation,^31–33^ we further analyzed the evolution of potential energy profiles over the time course of the MD simulations, and the results are presented in Figure S2 (Supporting Information). The potential energy vs time curve for each complex was stable, suggesting the stability of the simulated system.^34^ We also calculated the cosine content for the first three principal components (PCs; PC1, PC2, and PC3) for each class IIa HDAC-MEF2 complex. The results are presented in Figure S3 (Supporting Information). In shorter simulations, due to insufficient convergence, initial PCs exhibit the shape of the cosine function.^35,36^ Therefore, cosine content that measures the closeness of the PC to a cosine shape can be used to assess the convergence of the MD simulation.^36–38^ The value of cosine content close to 1 for the first few PCs does not correspond to the convergence of a simulation system.^35–38^ As shown in Figure S3, the average cosine content value for each class IIa HDAC-MEF2 complex is sufficiently lower than 1. For complexes with comparatively higher cosine content values for PC1, we removed highly flexible terminal portions (away from the binding interface) in HDACs and MEF2s and recalculated the cosine content values, which were found to be smaller than those calculated for the corresponding complete structures. Stable RMSD, *R*_g_, and potential energy profiles together with lower cosine content values for PC1, PC2, and PC3 as compared to 1 collectively suggest that our simulation systems are better stable and converged to produce a reliable prediction of interactions.

### 2.2 Contribution of Hydrophobic Interactions for the Formation of HDAC-MEF2 Complexes

We analyzed the binding interface between class IIa HDACs and MEF2s in each class IIa HDAC-MEF2 complex and found that there are consistent hydrophobic interactions that are responsible for the formation of these complexes. Table 1 lists pairs of residues that are responsible for the hydrophobic interactions. These residues are the common residues that we found from analyses of all three all-atom MD simulation trajectories of each complex. We found that L66 and L67 in all MEF2s consistently establish hydrophobic interactions with different class IIa HDACs. These residues are also in the list of residues that were predicted to establish hydrophobic interactions between HDAC7 and MEF2A in our recent report.^29^

**Table 1:** Hydrophobic Residues in HDAC-MEF2 Complexes

		HDACs				
MEF2s			HDAC4	HDAC5	HDAC7	HDAC9
Name	Chain	AA	AA	AA	AA	AA
MEF2A	A	M62	A167			
		L66, L67	V171			L151
		L66	L175			L147
		L67		L191		
	B	L66	L175	L187		L147
		L67	V179			V143
MEF2B	A	L66	L175	L187		L147
		L67	V179, L180			
		L66, L67		L191		V143
	B	L66	L175	L187	L89	L147
		L67	V171	V183		L151
MEF2C	A	L66	L175	L187, L191		V143, L147
		L67	V179			
		L66, L67			V85	
	B	L66	L175	L187	L89, I93	L147, L151
		L67	V171			
		L66, L67		V183		
		P75				A137
MEF2D	A	L66	L175	L187	L89	L147
		L67	V171	L192		
		L66, L67		L191	V85	
	B	L66	L175	L187	L89	L147
		L67			I93	
		L66, L67		V183		L151

Figure 3A shows that all residues in both chains A and B of all MEF2s that we predicted to establish hydrophobic interactions with class IIa HDACs are fully conserved in all MEF2s. Table S1 (Supporting Information) lists the amino acid residues in both class IIa HDACs and MEF2s in the complexes with available crystal structures. Besides the A167­(HDAC4)-M62­(MEF2A) residue pair, V171-L67, L175-L66, and V179-L67 interacting pairs were found in both predicted and crystal structures of HDAC4-MEF2A complexes. Moreover, V171-L67 and L175-L66 interacting pairs were the same in both predicted and crystal structures of HDAC4-MEF2D; L187-L66, L191-L66/L67, and V183-L66/L67 in HDAC5-MEF2D; L89-L66 and V85-L66/L67 in HDAC7-MEF2D; and L147-L66 and L151-L66/L67 in HDAC9-MEF2D.

**Figure 3:**
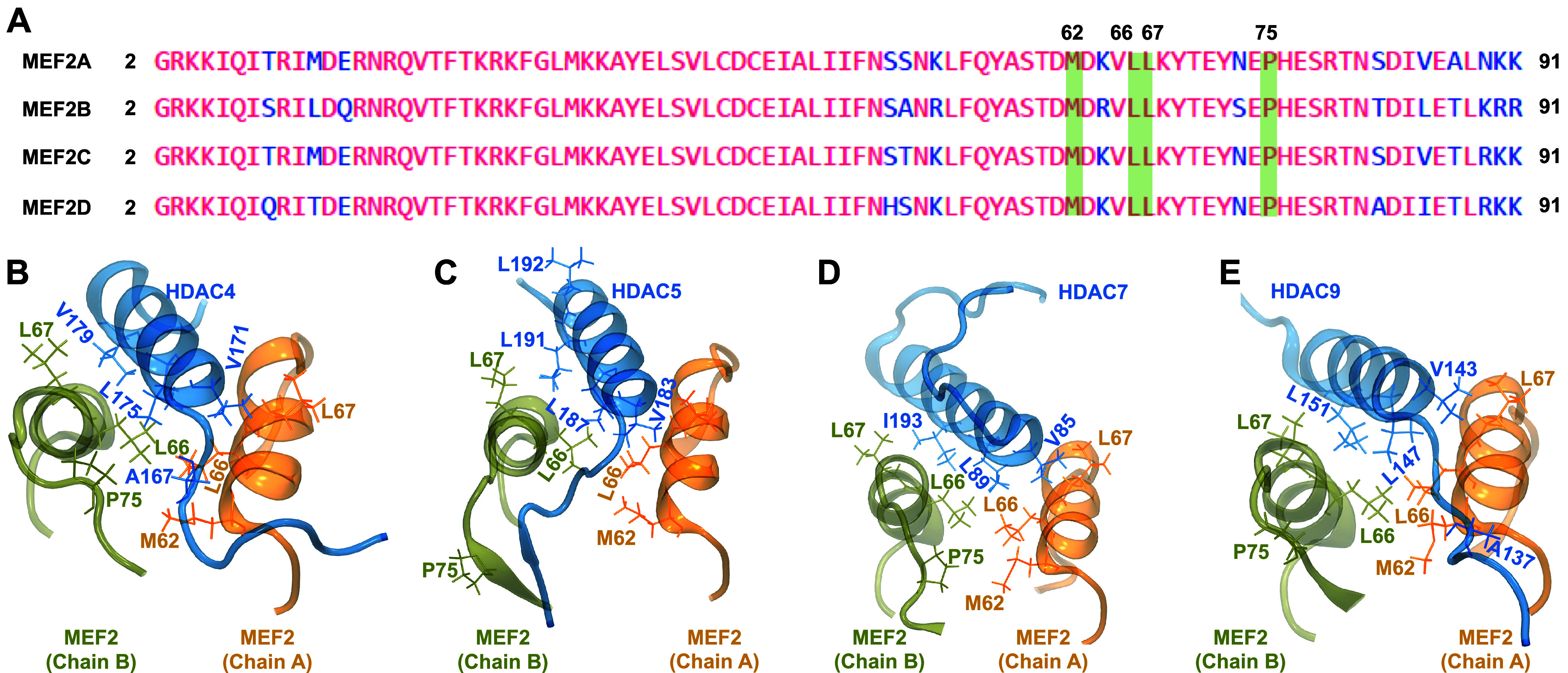
(A) Multiple sequence alignment of all MEF2s. (B) Locations of all amino acid residues in both class IIa HDACs and MEF2s that establish hydrophobic interactions in representative class IIa HDAC-MEF2 complexes. HDACs and MEF2 proteins are zoomed-in near the binding interface to highlight the locations of the interacting residues.

Notably, only amino acid residues from G169 to N181 in HDAC4, from W178 to S193 in HDAC5, from G83 to K96 in HDAC7, and from G139 to K154 in HDAC9 are available in the crystal structures for complexes of class IIa HDACs with MEF2D, in contrast to residues from G161 to K184 in HDAC4, residues from S173 to K196 in HDAC5, residues from K76 to N108 in HDAC7, and residues from G133 to K158 in HDAC9 that we used in our corresponding predicted complexes. Table S2 shows the structural differences in class IIa HDACs between the available crystal structure and the predicted complex for each system. The amino acid residues in bold and underlined letters in predicted structures represent the ones that fall within the same range of amino acids in the available crystal structures. Since other amino acid residues are far away from the MEF2A interaction range, we only selected the residues from position K145 in the HDAC4-MEF2A crystal structure for subsequent simulations. We also noticed that a few amino acids in other crystal structures are different from the sequences that were used to predict the corresponding complex. These structural differences might be a reason for a few discrepancies in the list of residue pairs that form hydrophobic interactions. Nevertheless, L66 and L67 in all MEF2s were found to be common in all simulations. This observation provides confidence in the prediction of complexes in our study. Figure 3B represents the positions of all amino acid residues in both class IIa HDACs and MEF2s listed in Table 1 in the 3D structure of the class IIa HDAC-MEF2 complexes. We used only one representative MEF2 (instead of all four MEF2s) to locate the position of amino acid residues, since the interacting residues are conserved.

### 2.3 Contribution of Hydrogen Bonding and Salt Bridges

Our analysis of hydrogen bonding between class IIa HDACs and MEF2s in each pair shows that hydrogen bonding moderately contributes to the formation of the class IIa HDAC-MEF2 complexes. Our analysis predicted that D63 and T70 in all MEF2s consistently form hydrogen bonds with residues in class IIa HDACs. Table 2 shows the residues in both class IIa HDACs and MEF2s that form hydrogen bonds with occupancies.

**Table 2:** Amino Acid Residues Responsible for the Formation of Hydrogen Bonding in Class IIa HDAC-MEF2 Complexes with Hydrogen Bonding Occupancies^a^

			HDACs							
MEF2s			HDAC4		HDAC5		HDAC7		HDAC9	
Name	Chain	AA	AA	% occupancy	AA	% occupancy	AA	% occupancy	AA	% occupancy
MEF2A	A	D63	S168	66.0 ± 10.4						
		T70			Q188	26.0 ± 8.0				
	B	T70	Q176	52.0 ± 3.0						
MEF2B	A	**D63**			S180	69.1 ± 7.3			**K154**	57.1 ± 13.5
		T70	Q176	32.3 ± 7.5						
MEF2C	A	D63					S82	59.6 ± 4.3		
		T70	Q176	30.2 ± 5.9						
MEF2D	A	D63					S82	90.7 ± 15.3		
	B	**D63**					**K96**	56.8 ± 4.4		
		T70	Q176	27.7 ± 6.3						

a The occupancy values are listed as mean ± standard deviation (s.d.) from three independent runs of each complex. The residues in the bold text are also predicted to establish salt bridges

Figure 4 shows the hydrogen bonding distances between atoms in different residues in both class IIa HDACs and MEF2s. Measurements from three independent simulations (same light colors) and their average values (corresponding dark colors) are presented. We found that some hydrogen bonds are intermittent, as shown in Figure 4D. As shown in Table 2, our analysis did not predict hydrogen bond formation between all class IIa HDACs and MEF2s, unlike all class IIa HDACs showing hydrophobic interactions with different MEF2s. Table S3 in the Supporting Information shows a list of amino acid residues that establish hydrogen bonds between residues in class IIa HDACs and MEF2s with crystal structures, and Figure S4 shows distance-time plots for corresponding hydrogen bonds. While residues establishing hydrogen bonds in HDAC4-MEF2A and HDAC4-MEF2D in crystal structures are also included in the results for corresponding predicted complexes, there are inconsistencies in the results for HDAC7-MEF2D and HDAC9-MEF2D structures in both crystals and predicted complexes that form hydrogen bonds. These inconsistencies are likely due to structural differences. Notably, the range of amino acids in class IIa HDACs are longer in our predicted complexes as compared to what was found in the crystal structures of class IIa HDACs with MEF2D.

**Figure 4:**
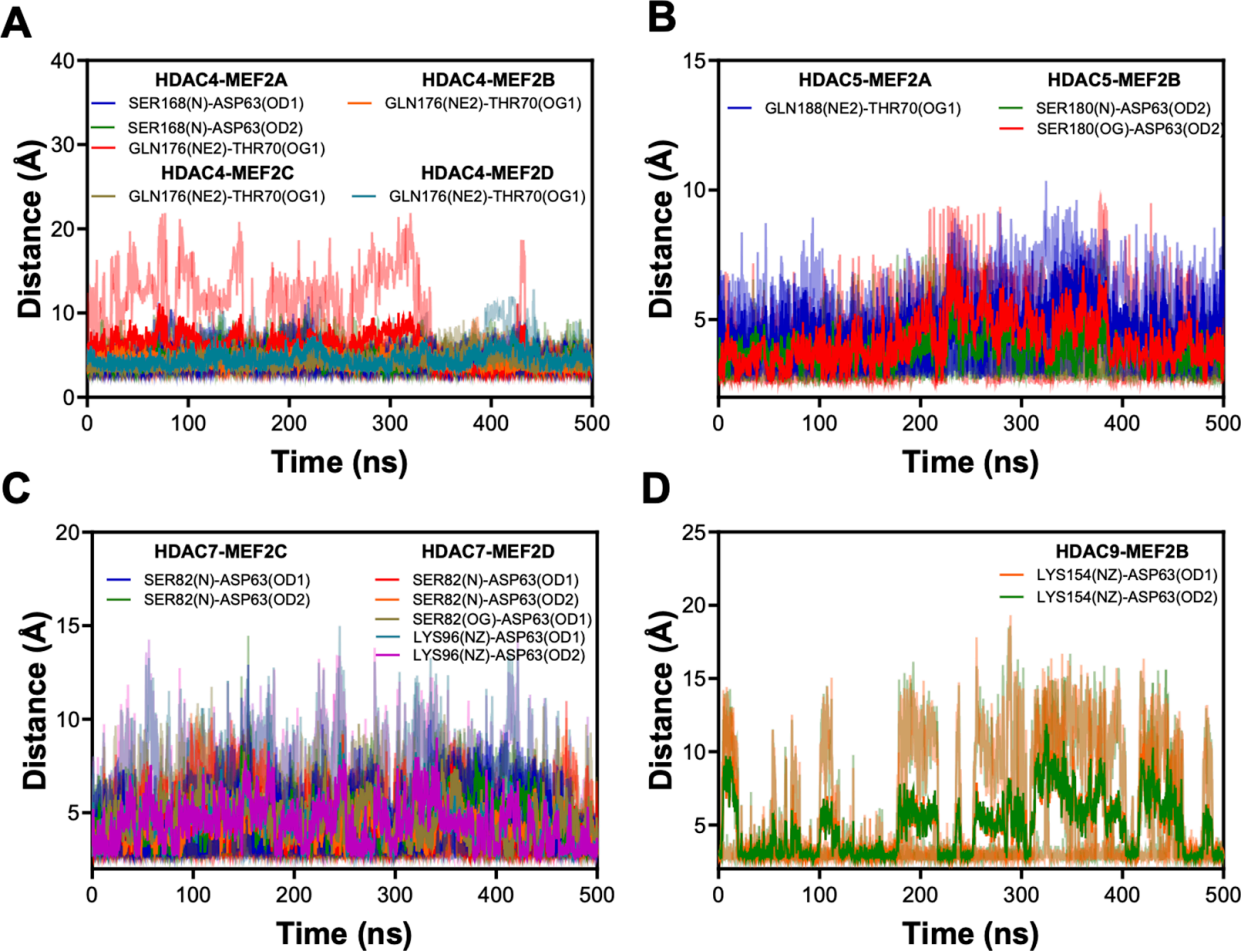
(A–D) Distance–time plots for hydrogen bonds between different amino acid residues in class IIa HDACs and MEF2s. The same light-colored data correspond to the measurement from three different runs with the respective dark color as the average values for each triplicate.

The residues in bold text (Table 2) that form hydrogen bonds between K96 in HDAC7 and D63 in MEF2D, as well as K154 in HDAC9 and D63 in MEF2B, also formed salt bridges for the predicted complexes, as shown in Figure S5. This observation reinforces the importance of these residues in the corresponding complex formations. Our analysis did not predict any salt bridges for the crystal structures, likely due to structural differences. The predicted salt bridges are quite few as compared to hydrogen bonds. Even though we predicted more hydrogen bonding than salt bridges, the hydrogen bonds are not as many as predicted hydrophobic interactions, suggesting that hydrogen bonding and salt bridges are not the main interaction mechanisms of the complex formations between class IIa HDACs and MEF2s. Figure 5 shows the location of amino acid residues that form hydrogen bonds in the 3D structures of class IIa HDAC-MEF2 complexes. Figure 5A shows that the amino acid residues in MEF2s that establish hydrogen bonding with different class IIa HDACs are conserved in all MEF2s. We, therefore, used only a representative MEF2 structure in complex with different HDACs.

**Figure 5:**
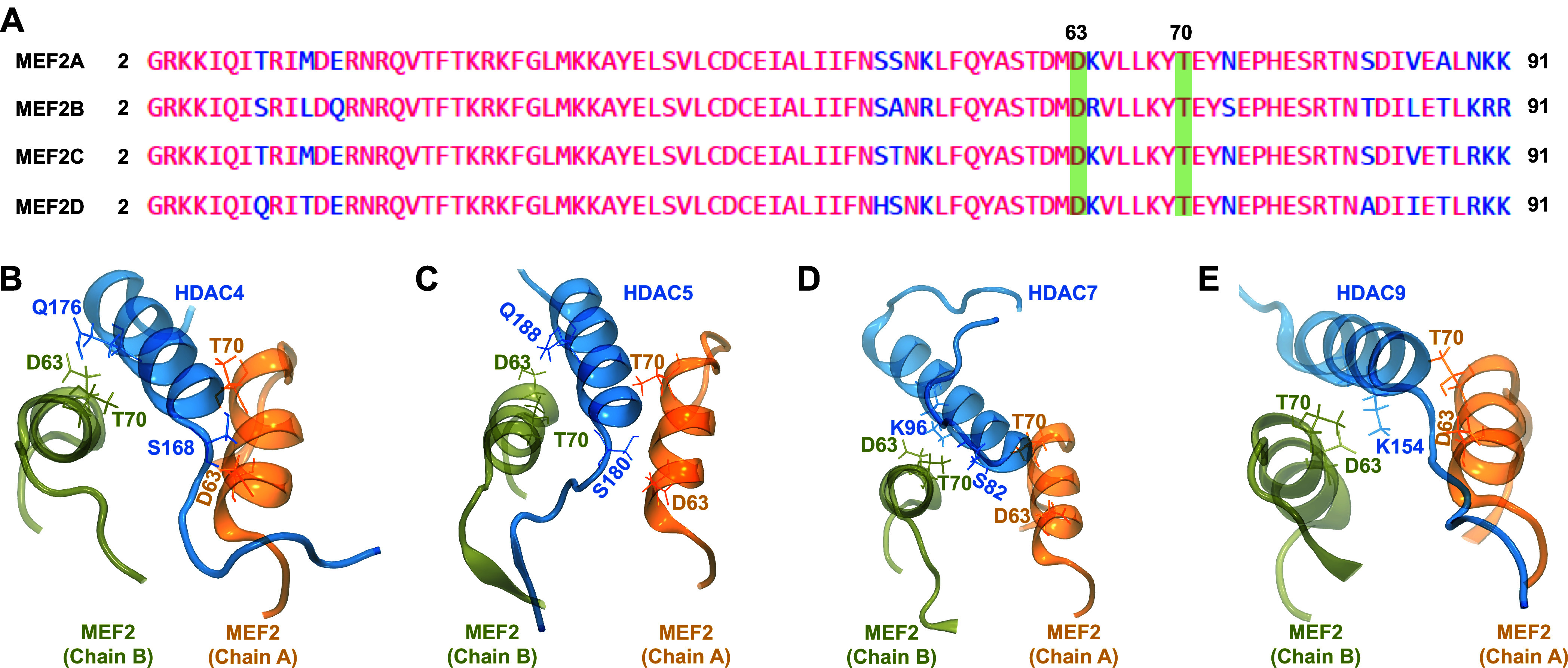
(A) Multiple sequence alignment of all MEF2s. (B) Locations of all amino acid residues in both class IIa HDACs and MEF2s that establish hydrogen bonds and a salt bridge in representative class IIa HDAC-MEF2 complexes. HDACs and MEF2 proteins are zoomed near the binding interface to highlight the locations of the interacting residues.

### 2.4 Binding Affinities of Class IIa HDAC-MEF2 Interactions

We calculated MM/GBSA binding free energies for all 15 predicted complexes and 5 crystal structures that we studied to quantify binding affinities between the class IIa HDACs and MEF2s. Even though the MM/GBSA binding free energy values are generally overestimated, the binding free energy values still offer a good comparison of binding affinities among different complexes.^39^ The MM/GBSA binding free energies in Table 3 show that the class IIa HDAC-MEF2 complexes bind with a comparable affinity. The same is true for the HDAC7-MEF2A complex that we recently reported.^29^ Table S4 (Supporting Information) lists the MM/GBSA binding free energy values for complexes with crystal structures.

**Table 3:** Binding Free Energies Calculated Using the MM/GBSA Approach Listed as Mean ± Standard Deviation from the Three Independent Runs of Each Complex

MEF2s	HDACs			
Name	HDAC4 Δ*G* (kcal/mol)	HDAC5 Δ*G* (kcal/mol)	HDAC7 Δ*G* (kcal/mol)	HDAC9 Δ*G* (kcal/mol)
**MEF2A**	–51.6 ± 3.5	–48.9 ± 3.2		–49.8 ± 4.4
**MEF2B**	–54.7 ± 7.1	–52.3 ± 4.5	–51.7 ± 8.3	–49.8 ± 4.6
**MEF2C**	–54.1 ± 7.2	–48.8 ± 7.3	–45.6 ± 2.1	–48.9 ± 2.5
**MEF2D**	–49.3 ± 6.9	–49.1 ± 6.0	–53.3 ± 8.5	–49.8 ± 5.7

We observed very similar affinity values for both predicted and the complex with the available crystal structure for the HDAC4-MEF2A complex. However, the binding affinities for other complexes with crystal structures are weaker than the values for the corresponding structures we predicted. We obtained similar affinity (MM/GBSA binding free energy) values when we ignored extra amino acids from the simulation trajectories in our predicted complexes to match the amino acid residues available in the crystal structures. This implies that the weaker affinities for these crystal structures are due to the structural differences (fewer available class IIa HDACs amino acid residues). We also performed interfacial contact analysis, and the results are presented in Table S5 (Supporting Information). As shown in Table S5, the number of interfacial contacts for the crystal structures involving MEF2D was much lower compared to the corresponding predicted structures. The higher number of interfacial contacts corresponds to a stronger affinity.^40^ The results from the interfacial contact analysis thus support weaker affinities for the crystal structures with MEF2D as compared to the corresponding predicted structures. We found a similar number of contacts for both the crystal and predicted complexes of HDAC4-MEF2A. In the simulation of HDAC4-MEF2A crystal structure, we did not use HDAC4 amino acid residues that reside along a long helical chain and are far away from the MEF2A interacting site. This truncation offered a shorter simulation time. However, we still included a slightly longer range (G145 to K183) in the crystal as compared to the corresponding predicted structure (G161 to K184). The affinity of the complex formation was found to be −48.6 ± 2.3 kcal/mol (Table S4, Supporting Information), which is very similar to what we calculated for the corresponding predicted complex. Moreover, analyses of binding interactions (Tables S1 and S3, Supporting Information) also did not predict any residues in HDAC4 outside the range used in the predicted complex. This justifies the truncation of the HDAC4-MEF2A crystal structure for simulations.

### 2.5 Surface Plasmon Resonance (SPR) Validations

The SPR-based technique is very useful to validate direct binding between biomolecules investigated using another method.^41–45^ Since all amino acid residues in MEF2s that are involved in hydrophobic interactions (Figure 3) and hydrogen bonding, as well as salt bridges (Figure 5), are conserved in all MEF2s, we selected MEF2A as a representative MEF2 and performed direct binding experiments with all four class IIa HDACs. This significantly reduced our experimental cost. We repeated SPR experiments three times for each complex formation.

Figure 6 shows representative SPR sensorgrams for the direct binding of class IIa HDACs to MEF2A immobilized onto CM5 chip surfaces. We injected each concentration of all of the class IIa HDACs in duplicate. The continuous colored lines shown in Figure 6 correspond to experimental data. We fitted the experimental data to the 1:1 kinetic binding model. The dotted lines in Figure 6 represent the fits. The association rate constant (*k*_a_), dissociation rate constant (*k*_d_), and equilibrium dissociation constant (*K*_D_, affinity) values derived via SPR data fitting are provided in Figure 6. The *k*_a_, *k*_d_, and *K*_D_ values are listed as mean ± standard deviation from three independent experiments. The K_D_ values for each class IIa HDAC-MEF2A complex are fairly comparable to nanomolar affinity. Comparable MM/GBSA binding free energy values listed in Table 3 agree with the fairly comparable nanomolar affinities as obtained using SPR. A direct comparison of the MM/GBSA affinity value with the corresponding SPR affinity is not recommended. The discrepancy arises due to technical differences in the two methods. In MD simulations, the complex moves freely in an aqueous cubic box. In SPR experiments, one of the binding partners (MEF2A in our case) is restricted to the chip surface, and hence, the complex formed as a result of binding. Nevertheless, the similarity of binding affinities in SPR experiments validates the similarity in MM/GBSA values as observed in MM/GBSA calculations. Slightly higher SPR affinities (lower *K*_D_ values) for HDAC4-MEF2A and HDAC7-MEF2A direct bindings might correspond to a larger number of hydrogen bonds formed for these complexes, as shown in Table 2 and in our previous report.^29^ There were two pairs of amino acid residues predicted for the HDAC7-MEF2A complex that establish hydrogen bonding.^29^ Moreover, the lack of hydrogen bonds HDAC9-MEF2A in Table 2 as compared to other class IIa HDACs might correspond to slightly weaker affinity (higher experimental *K*_D_ value) for the HDAC9-MEF2A complex formation. Altogether, these SPR experiments not only validated the class IIa HDAC-MEF2A binding experimentally but also provided confidence in our computational results.

**Figure 6:**
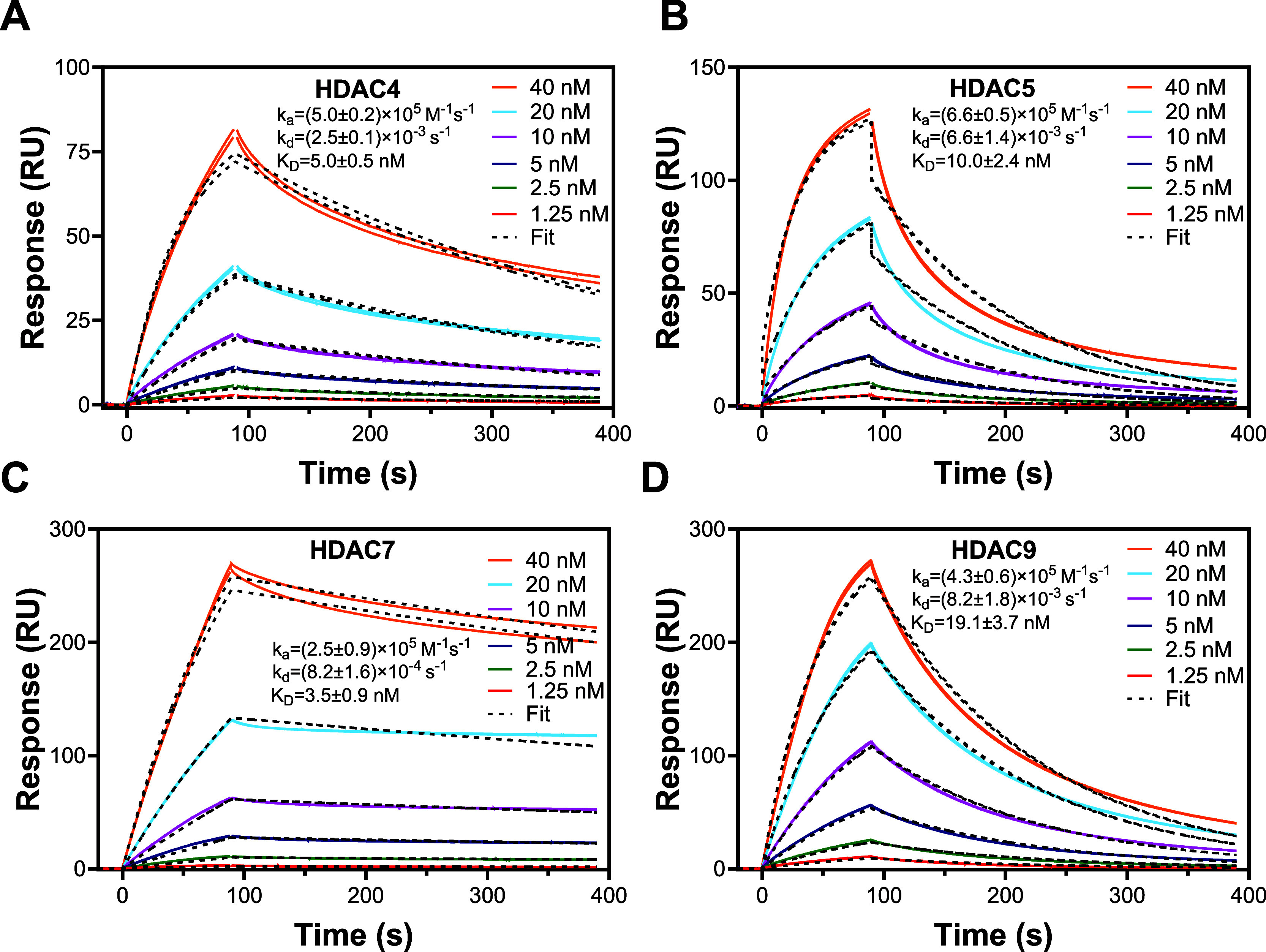
Representative SPR Sensorgrams for direct bindings of (A) HDAC4, (B) HDAC5, (C) HDAC7, and (D) HDAC9 to MEF2A. MEF2A was immobilized onto CM5 chips. The continuous colored lines are experimental data and the black dotted lines are fit to the 1:1 kinetics binding model. Each concentration of all class IIa HDACs were injected in duplicate.

## 3 Materials and Methods

### 3.1 Proteins, Sensorchips, and Reagents

HDAC4 (catalog no. MBS953633), HDAC5 (catalog no. MBS388238), and HDAC9 (catalog no. MBS2030545) were purchased from MyBioSource (San Diego, CA). HDAC7 (catalog no. LS-G22737) and MEF2A (catalog no. LS-G29304) were purchased from LSBio (Newark, CA). Series S CM5 sensor chips (catalog no. 29149603), amine coupling kit (catalog no. BR100050), and HBS-P+ buffer (catalog no. BR100671) were purchased from Cytiva (Marlborough, MA).

### 3.2 System Preparation

UniProt^46^ FASTA sequences (accession ID P56524 for HDAC4, accession ID Q9UQL6 for HDAC5, accession ID Q8WUI4 for HDAC7, accession ID Q9UKV0 for HDAC9, accession ID Q02078 for MEF2A, accession ID Q02080 for MEF2B, accession ID Q06413 for MEF2C, and accession ID Q14814 for MEF2D) corresponding to all class IIa HDACs and MEF2s were used to predict 15 different complexes of class IIa HDACs (HDAC4, HDAC5, HDAC7, and HDAC9) with all four MEF2s (4 HDAC4-MEF2 complexes, 4 HDAC5-MEF2 complexes, 3 HDAC7-MEF2 complexes, and 4 HDAC9-MEF2 complexes) using AlphaFold Colab.^47^ We have investigated the remaining HDAC7-MEF2A complex in our recent report.^29^ We supplied the entire sequence of class IIa HDACs and MEF2s amino acids from positions 2 to 91 as input sequences during the generation of the full-length complex structures. Based on these results and multiple sequence alignments in a recently published report,^29^ we chose the truncated region of all class IIa HDACs in complex with all MEF2s for all-atom simulations. We also used PDB ID 7XUZ^48^ (HDAC4-MEF2A), PDB ID 8PDE^49^ (HDAC4-MEF2D), PDB ID 8Q9P^49^ (HDAC5-MEF2D), PDB ID 8Q9Q^49^ (HDAC7-MEF2D), and PDB ID 8Q9R^49^ (HDAC9-MEF2D) for all-atom MD simulations of published crystal structures containing human HDACs and human MEF2s.

### 3.3 MD Simulations

We carried out all-atom MD simulations of each individual complex utilizing the NAMD software^50^ (version 2.14 or 3.0) and CHARMM36m^51^ force field as we used in our prior investigations.^29,39,41–43^ We prepared the simulation input files using the CHARMM-GUI Web server.^52^ Cubic boxes filled with TIP3 water model were used for solvation with the addition of 150 mM NaCl for neutralization. The size of the solvated cubic boxes for all 20 different systems varied from 73 × 73 × 73 Å^3^ with a total number of atoms of 36,118 to 92 × 92 × 92 Å^3^ with a total number of atoms of 72,803. We then minimized the solvated and ion-neutralized systems for 10,000 steps with heavy atoms restrained harmoniously, followed by 100 ps equilibration under the NVT ensemble with 1 fs time step at 300 K. The long-range interactions were calculated using the Particle Mesh Ewald (PME) method.^53^ The nonbonded interactions were cut off at 12 Å. We executed production runs for analysis using a time step of 2 fs for 500 ns at 300 K, 1 atm pressure, and Langevin dynamics with a damping constant of 1 ps^–1^ under the NPT conditions. We conducted three independent replica runs for each complex. Root-mean-square deviation (RMSD), radius of gyration (*R*_g_), potential energy measurements, and cosine content calculations were performed to assess the stability and convergence of MD simulation trajectories.

### 3.4 Surface Plasmon Resonance (SPR)

All SPR measurements were conducted using a Biacore T200 instrument (Marlborough, MA) with CM5 chips at 25 °C. MEF2A proteins were diluted in 10 mM sodium acetate buffer (pH 5.0) and immobilized onto the CM5 chips using standard amine coupling chemistry. A neighboring flow cell (FC) was activated and deactivated using the same surface chemistry as the FC used to immobilize MEF2A, but no proteins were immobilized onto the reference FC. HBS-P (10 mM Hepes pH 7.4, 150 mM NaCl, 0.05% surfactant P20), which was 10× diluted from HBS-P+, was used as the immobilization running buffer (buffer that runs in the background during immobilization). Different concentrations of class IIa HDACs (40–1.25 nM, 2-fold dilutions) were prepared in kinetics buffer (buffer that was used to dilute the class IIa HDACs as well as runs in background during binding) over the reference and MEF2A immobilized surfaces. Each concentration of all class IIa HDACs was injected in duplicate to monitor technical reproducibility. HBS-P was used as the kinetics running buffer for binding of HDAC7 and HDAC9 to MEF2A. HBS-P supplemented with 1% (v/v) glycerol was used as the kinetics buffer for binding of HDAC4 and HDAC5 to MEF2A to avoid glycerol mismatch that was included in the storage buffer of HDAC4 and HDAC5. A flow rate of 30 μL/min was maintained during injections of all class IIa HDACs. The contact and dissociation times used for class IIa HDAC-MEF2A bindings were 90 and 300 s, respectively. A 20 s pulse of a regeneration solution containing 1:500 H_3_PO_4_ (H_3_PO_4_:ddH_2_O, v/v) was injected for surface regeneration. All SPR sensorgrams obtained for analysis were both blank (buffer only response) and reference (response corresponding to the reference FC) subtracted. All SPR experiments were repeated in three independent experiments.

### 3.5 Data Analysis

The Visual Molecular Dynamics (VMD) software^54^ was used to analyze all MD simulation trajectories, visualize complex structures, root-mean-square deviation (RMSD) measurements, interfacial contact analysis, and generate 3D figures of complexes. Measurements of radius of gyration (*R*_g_) and PCA-based cluster analysis were carried out using Carma.^55^ The NAMD Energy plugin in VMD was used to calculate the potential energy. The MDAnalysis tool was used to calculate cosine contents.^36,56^ NAMD was used to calculate binding free energies using the MM/GBSA method^57^ and to compare binding affinities using a simplistic approach, as we adopted for the HDAC7-MEF2A complex in our recent publication.^29^ A 2.8 Å distance cutoff was used in VMD to analyze hydrophobic interactions, 3.5 Å distance and 30° angle cutoffs for hydrogen bonding, and a 3.5 Å distance cutoff for salt bridges and interfacial contacts. COBALT^58^ was used in multiple sequence alignments. Biacore T200 evaluation software version 3.2.1 (Marlborough, MA) was used to fit SPR sensorgrams using a 1:1 kinetics binding model. GraphPad Prism (Boston, MA) was used to plot the graphs.

## 4 Conclusions

MEF2 transcription factors take part in the differentiation as well as the survival of neurons in the central nervous system. Class IIa HDACs regulate several cellular processes, including cell differentiation, proliferation, and apoptosis, via their interactions with MEF2s. This physical interaction of class IIa HDACs with MEF2s is associated not only with myogenesis but also with neuronal survival and axon branching. We present a comprehensive investigation to map interactions among all class IIa HDACs and MEF2s. We predicted key amino acid residues that are responsible for establishing the class IIa HDAC-MEF2 complexes. Our analysis showed that hydrophobic interactions play an important and consistent role in the formation of the class IIa HDAC-MEF2 complex as compared to hydrogen bonding and salt bridges. We predict that L66 and L67 in all MEF2s contribute mostly to consistent hydrophobic interactions. All predicted residues that establish hydrophobic interactions, hydrogen bonding, and salt bridges are conserved in all MEF2s. Moreover, the class IIa HDAC-MEF2 complexes exhibit comparable binding affinities, as evidenced by comparable MM/GBSA binding free energy values. SPR-based experiments not only validate the complex formations among all class IIa HDACs and MEF2A with fairly comparable nanomolar affinity (3.5 nM to 19.1 nM) but also render confidence in our computational results. Our investigation offers the scientific community valuable insights to further understand, explore, characterize, and investigate biomolecular systems that include the class IIa HDAC-MEF2 complex formations. Furthermore, inhibition of class IIa HDAC-MEF2 complex formation can help develop new therapeutics to combat neurodegenerative diseases. Therefore, in the absence of all crystal structures, our investigation is valuable for future research endeavors along this avenue.

## Supplementary Material













## Data Availability

The PDB files of all 15 predicted complexes at the end of 500 ns all-atom MD simulations, topology and parameter files, configuration files, and other files including coordinate (.pdb) and structure (.psf) files, along with other files prepared for crystal structures used in simulations, are provided as the supporting files. Coordinate and structure input files for other complexes used in this study can be obtained from the corresponding author upon request. PDB structures 7XUZ, 8PDE, 8Q9P, 8Q9Q, and 8Q9R are available in the Protein Data Bank (https://www.rcsb.org/). Corresponding files for the CHARMM36m force field that were used in this study are available on the MacKerell Lab webpage (https://mackerell.umaryland.edu/charmm_ff.shtml) or on the CHARMM-GUI webpage (https://www.charmm-gui.org/). NAMD and VMD software can be downloaded from the developer’s webpage (https://www.ks.uiuc.edu/Development/). CARMA can be downloaded from the Glykos Lab webpage (https://utopia.duth.gr/glykos/Carma.html). COBALT multiple sequence alignment tool can be accessed through the National Center for Biotechnology Information (NCBI) webpage (https://www.ncbi.nlm.nih.gov/tools/cobalt/re_cobalt.cgi). Biacore T200 evaluation software version 3.2.1 comes with the Biacore T200 instrument and can be purchased from Cytiva (https://www.cytivalifesciences.com/). GraphPad Prism can be purchased from the GraphPad website (https://www.graphpad.com/).

## Notes

(Figure S1) Representative class IIa HDAC-MEF2 complex structures with PDB structures at the end of 500 ns MD simulations (Figure S2) RMSD and *R*_g_ measurements for all 15 predicted complexes as well as complexes with crystal structures; potential energy vs time plots for all complex structures simulated in this study; (Figure S3) cosine contents for all complex structures simulated in this study; (Figure S4) distance–time plots for hydrogen bonds between different amino acid residues in HDACs and MEF2s with crystal structures; (Figure S5) distance–time plots for salt bridges between different amino acid residues in predicted class IIa HDACs and MEF2s; (Table S1) hydrophobic residues in HDAC-MEF2 complexes with crystal structures; (Table S2) comparison of amino acid residues in the available crystal and corresponding predicted complex structures; (Table S3) amino acid residues responsible for the formation of hydrogen bonding in class IIa HDAC-MEF2 complexes and hydrogen bonding occupancies for complexes with crystal structures; (Table S4) MM/GBSA binding free energies listed as mean ± standard deviation (s.d.) from the three independent runs of each complex with crystal structures; (Table S5) comparison of the number of interfacial contacts between the predicted complexes and corresponding complexes with crystal structures (PDF)
PDB structures (ZIP)
Sample topology parameter files (ZIP)
Sample configuration files (ZIP)
Sample input files for crystals (ZIP)
Average structures (ZIP)
