# Supplementary material for: Mapping the Interactions Among Class IIa Histone Deacetylases and Myocyte Enhancer Factor 2s
Source: J Chem Inf Model. 2025 Jun 6;65(12):6249–60. doi: 10.1021/acs.jcim.5c00858 (PMC12199305; doi:10.1021/acs.jcim.5c00858)
Supplement: Supplementary file 1 [file ci5c00858_si_001.pdf]

Supporting Information

Mapping the Interactions Among Class IIa Histone Deacetylases and Myocyte Enhancer Factor 2s

Narayan Gautam[a, b], Sophia Wang[c], Aykut Üren[d], Prem P. Chapagain[e, f*], Narayan P. Adhikari[a*], and Purushottam B. Tiwari[d*]

[a]Central Department of Physics, Tribhuvan University, Kirtipur, Kathmandu 44613, Nepal

[b]Tri-Chandra Multiple Campus, Tribhuvan University, Ghantaghar, Kathmandu 44613, Nepal

[c]Department of Biology, Georgetown University, Washington, D.C. 20057, USA

[d]Department of Oncology, Georgetown University, Washington, D.C. 20057, USA

[e]Department of Physics, Florida International University, Miami, FL 33199, USA

[f]Biomolecular Sciences Institute, Florida International University, FL 33199, USA

*Corresponding Authors

P. P. Chapagain
Email: chapagap@fiu.edu
Tel: +1(305) 348 6266

N. P. Adhikari
Email: narayan.adhikari@cdp.tu.edu.np
Tel: +977(1) 433 1054

P. B. Tiwari
Email: pbt7@georgetown.edu
Tel: +1(202) 687 3841

S1. Class IIa HDAC-MEF2 structures, stability, and cosine contents

Figure 1 in the main text show representative structures of 15 class IIa HDAC-MEF2 complexes. Structure files of these complexes are provided in PDB format as a separate supporting document (PDB_structures, zip). We have also provided sample topology and parameter files as a separate supporting document (Sample_topology_parameter_files, zip) obtained from CHARMM-GUI web-server when we prepared simulation input files. Another separate supporting file that has configuration files that we used during equilibration, production, and data analysis (Sample_configuration_files, zip) is also provided. Another supporting file is also provided that has sample input files, including coordinates (.pdb), structures (.psf), and other files needed for simulations of the complexes with crystal structures (Sample_input_files_for_crystals, zip).

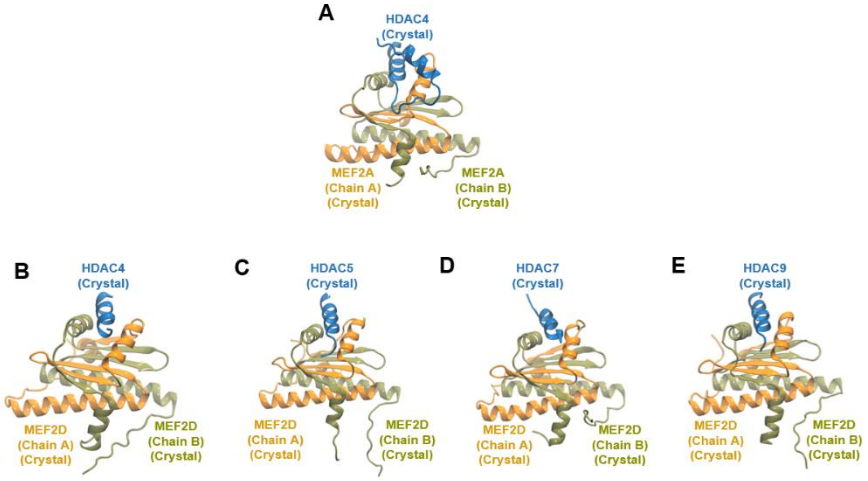

Figure S1. (A)-(E) Representative human class IIa HDAC-MEF2 complexes at 500 nM all-atom MD simulations of PDB structures. Structures in light blue color represent class IIa HDACs. Structures in light orange and light brown colors represent two monomers of the MEF2 dimers.

Figure S1 shows the human class IIa HDAC-MEF2 complexes at 500 ns all-atom MD simulations of PDB structures. We considered K145 to K183 in HDAC4 to prepare a HDAC4-MEF2A complex for simulations from the available HDAC4-MEF2A crystal structure. We also obtained the representative average structures from PCA-based cluster analysis as we did in our previous report[1] and the structures are provided as a separate document (Average_Structures.zip). For better clarity of structures, we recommend using psf and pdb files together that we obtained

after passing these average structures through “PDB Reader & Manipulator” in CHARMM-GUI webserver.[2]

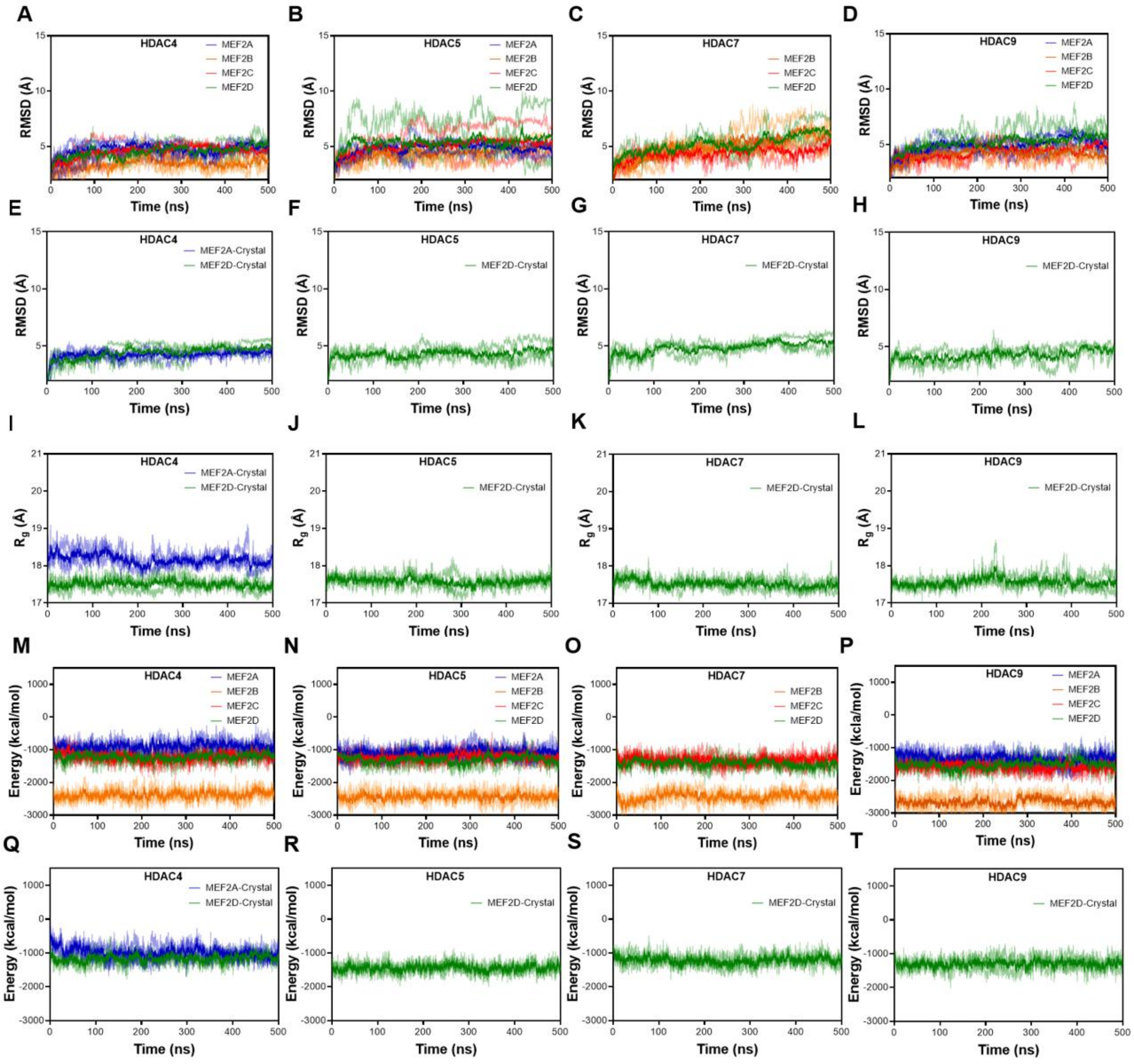

Figure S2. (A)-(D) Root mean square deviation (RMSD) measurements for predicted class IIa HDAC-MEF2 complexes. (E)-(H) RMSD and (I)-(L) radius of gyration (R_g) measurements for class IIa HDAC-MEF2 complexes with crystal structures. (M)-(P) Potential energy vs. time plots for predicted class IIa HDAC-MEF2 complexes. (Q)-(T) Potential energy vs. time plots for class IIa HDAC-MEF2 complexes with crystal structures. The same light-colored data correspond to

the measurement from three different runs with the respective dark color as the average values for each triplicate.

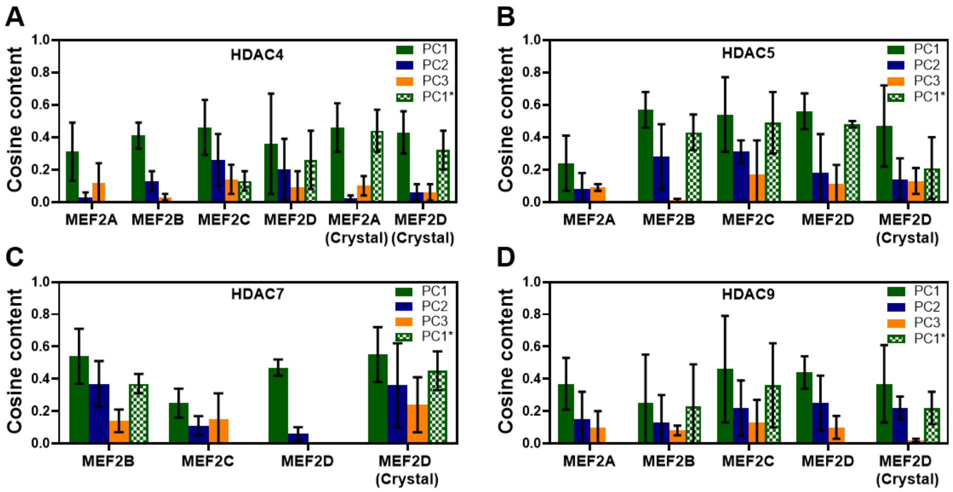

Figure S3. (A)-(D) Cosine content calculations for the first three principal components (PC1, PC2, and PC3) for all class IIa HDAC-MEF2 complexes that were simulated in this study. PC1* plots correspond to the cosine contents calculated for the same systems with highly flexible terminal loops removed. The colored bars represent mean and the error bars represent s.d. values calculated from the cosine contents determined from three independent replica runs for each system.

S2. Hydrophobic interactions of HDAC-MEF2 crystal structures and comparison of amino acid residues in available crystal and corresponding predicted complex structures

MEF2s			HDACs			
Name	Chain	AA	HDAC4	HDAC5	HDAC7	HDAC9
			AA	AA	AA	AA
MEF2A	A	L66	L175			
		L67	V179			
	B	L66	L175			
		L67	V171			
MEF2D	A	L66	V179	L187	L89	L147
		L67	L180			
		L66, L67		L191		V143
	B	L66	L175	L187	L89	L147
		L67	V171			L152
		L66, L67		V183	V85	L151

Table S1. Hydrophobic residues in class IIa HDAC-MEF2 complexes with crystal structures.

Table S2 shows the comparison of class IIa HDAC amino acid residues in crystals and corresponding predicted structures.

Complex structures		Class IIa HDAC sequences
HDAC4-MEF2A	Crystal (7XUZ, A)	145- KQHREQKLQQLKNKEKGKESAVASTEVKMKLQEFVLNKK-183
	Predicted	161-**GKESAVASTEVKMKLQEFVLNKKK**-184
HDAC4-MEF2D	Crystal (8PDE, C)	169-GEVKMKLQEFVLN-181
	Predicted	161-GKESAVAS**TEVKMKLQEFVLN**KKK-184
HDAC5-MEF2D	Crystal (8Q9P, X)	178-WGSGEVKLRLQEFLLS-193
	Predicted	173-SKESA**IASTEVKLRLQEFLLS**KSK-196
HDAC7-MEF2D	Crystal (8Q9Q, X)	83-GVVKQKLAEVILKK-96
	Predicted	75-SKRSAVAS**SVVKQKLAEVILKK**QQAA-100
HDAC9-MEF2D	Crystal (8Q9R, C)	139-GSGEVKQKLQEFLLSK-154
	Predicted	133-GRERAV**ASTEVKQKLQEFLLSK**ATK-158

Table S2. Comparison of amino acid residues in available crystal structures and that were used to predict different class IIa HDAC-MEF2 complexes. The amino acid residues in bold and underlined letters for predicted structures represent the ones that fall within the same range of amino acids in the available crystal structures. The amino acid residues before position K145 from the available crystal structure were not considered for the simulation of HDAC4-MEF2A crystal (main text). The texts inside parentheses for complex structures correspond to PDB ID and chain (PDB ID, chain) used in simulations. The letters in red represent differences in class IIa HDAC amino acids in the sequences of crystal and predicted structures.

S3. Hydrogen bonding and salt bridges in complexes with crystal structures

MEF2s (Crystal)			HDACs (Crystal)							
			HDAC4		HDAC5		HDAC7		HDAC9	
Name	Chain	AA	AA	% Occupancy	AA	% Occupancy	AA	% Occupancy	AA	% Occupancy
**MEF2A**	**B**	D63	S168	39.7±10.5				-		
**MEF2D**	**A**	T70	Q176	20.4±4.7						
	**B**	T70							Q148	27.2±0.9

Table S3. Amino acid residues responsible for the formation of hydrogen bonding in class IIa HDAC-MEF2 complexes and hydrogen bonding occupancies for complexes with crystal

structures. The occupancy values are listed as mean±standard deviation (s.d.) from the three independent runs of each complex.

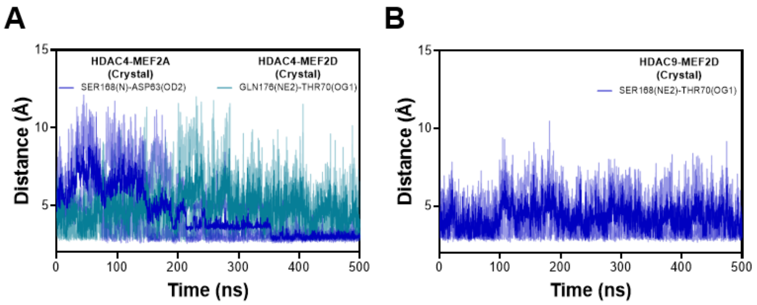

Figure S4. (A)-(B) Distance-time plots for hydrogen bonds between different amino acid residues in HDACs and MEF2s with crystal structures. The same light-colored data correspond to the measurement from three different runs with the respective dark color as the average values for each triplicate.

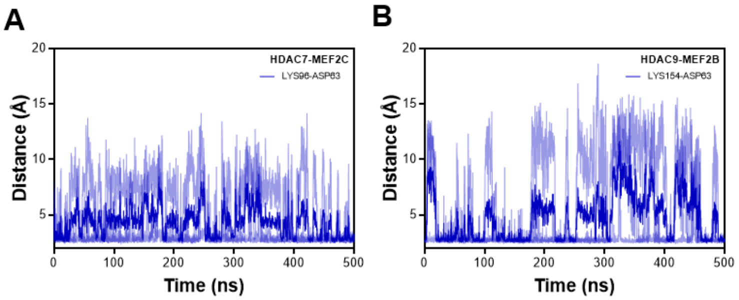

Figure S5. (A)-(B) Distance-time plots for salt bridges between different amino acid residues in predicted class IIa HDACs and MEF2s. The same light-colored data correspond to the measurement from three different runs with the respective dark color as the average values for each triplicate.

S4. Binding affinities for complexes with crystal structures

MEF2s	HDACs			
**Name**	**HDAC4 ΔG (kcal/mol)**	**HDAC5 ΔG (kcal/mol)**	**HDAC7 ΔG (kcal/mol)**	**HDAC9 ΔG (kcal/mol)**
**MEF2A**	-48.6±2.3	-		
**MEF2D**	-28.9±0.9 (-27.6±1.1)	-35.1±0.6 (-37.8±3.3)	-26.5±0.7 (-25.5±0.5)	-34.5±0.8 (-35.0±2.0)

Table S4. MM/GBSA binding free energies listed as mean±standard deviation (s.d.) from the three independent runs of each complex with crystal structures. The MM/GBSA values inside parentheses are calculated for modelled complex by removing extra residues from the complex to match amino acid residues as presented in the corresponding crystal structure.

S5. Comparison of number of interfacial contacts

MEF2s		HDACs			
**Name**		**HDAC4**	**HDAC5**	**HDAC7**	**HDAC9**
**MEF2A**	Predicted	71.0±6.1	-		
	Crystal	62.0±6.1			
**MEF2D**	Predicted	69.3±2.9	67.3±5.0	77.3±7.0	75.7±4.9
	Crystal	35.7±1.5	40.7±3.2	38.3±4.2	36.7±0.6

Table S5. Comparison of number of interfacial contacts between the predicted complexes and corresponding complexes with crystal structures. The number of contact values are listed as mean±standard deviation (s.d.) from the three independent runs of each complex.

REFERENCES

1. Tiwari, P. B.; Chapagain, P. P.; Uren, A. Investigating molecular interactions between oxidized neuroglobin and cytochrome c. *Sci. Rep.* **2018**, *8* (1), 10557.

2. Lee, J.; Cheng, X.; Swails, J. M.; Yeom, M. S.; Eastman, P. K.; Lemkul, J. A.; Wei, S.; Buckner, J.; Jeong, J. C.; Qi, Y.; et al. CHARMM-GUI Input Generator for NAMD, GROMACS, AMBER, OpenMM, and CHARMM/OpenMM Simulations Using the CHARMM36 Additive Force Field. *J. Chem. Theory. Comput.* **2016**, *12* (1), 405-413.
